# Longitudinal Alterations in Gait Features in Growing Children With Duchenne Muscular Dystrophy

**DOI:** 10.3389/fnhum.2022.861136

**Published:** 2022-06-02

**Authors:** Ines Vandekerckhove, Marleen Van den Hauwe, Nathalie De Beukelaer, Elze Stoop, Marije Goudriaan, Margaux Delporte, Geert Molenberghs, Anja Van Campenhout, Liesbeth De Waele, Nathalie Goemans, Friedl De Groote, Kaat Desloovere

**Affiliations:** ^1^Department of Rehabilitation Sciences, KU Leuven, Leuven, Belgium; ^2^Department of Child Neurology, University Hospitals Leuven, Leuven, Belgium; ^3^Clinical Motion Analysis Laboratory, University Hospitals Leuven, Leuven, Belgium; ^4^Department of Human Movement Sciences, Vrije Universiteit Amsterdam, Amsterdam, Netherlands; ^5^Interuniversity Institute for Biostatistics and Statistical Bioinformatics (I-BIOSTAT), KU Leuven, Leuven, Belgium; ^6^Interuniversity Institute for Biostatistics and Statistical Bioinformatics (I-BIOSTAT), Data Science Institute, Hasselt University, Hasselt, Belgium; ^7^Department of Development and Regeneration, KU Leuven, Leuven, Belgium; ^8^Department of Orthopedics, University Hospitals Leuven, Leuven, Belgium; ^9^Department of Movement Sciences, KU Leuven, Leuven, Belgium

**Keywords:** Duchenne muscular dystrophy, typically developing children, three-dimensional gait analysis, gait pattern, longitudinal study, mixed models for repeated measures

## Abstract

Prolonging ambulation is an important treatment goal in children with Duchenne muscular dystrophy (DMD). Three-dimensional gait analysis (3DGA) could provide sensitive parameters to study the efficacy of clinical trials aiming to preserve ambulation. However, quantitative descriptions of the natural history of gait features in DMD are first required. The overall goal was to provide a full delineation of the progressive gait pathology in children with DMD, covering the entire period of ambulation, by performing a so-called mixed cross-sectional longitudinal study. Firstly, to make our results comparable with previous literature, we aimed to cross-sectionally compare 31 predefined gait features between children with DMD and a typically developing (TD) database (1). Secondly, we aimed to explore the longitudinal changes in the 31 predefined gait features in growing boys with DMD using follow-up 3DGA sessions (2). 3DGA-sessions (*n* = 124) at self-selected speed were collected in 27 boys with DMD (baseline age: 4.6–15 years). They were repeatedly measured over a varying follow-up period (range: 6 months–5 years). The TD group consisted of 27 children (age: 5.4–15.6 years). Per measurement session, the spatiotemporal parameters, and the kinematic and kinetic waveforms were averaged over the selected gait cycles. From the averaged waveforms, discrete gait features (e.g., maxima and minima) were extracted. Mann-Whitney *U* tests were performed to cross-sectionally analyze the differences between DMD at baseline and TD (1). Linear mixed effect models were performed to assess the changes in gait features in the same group of children with DMD from both a longitudinal (i.e., increasing time) as well as a cross-sectional perspective (i.e., increasing baseline age) (2). At baseline, the boys with DMD differed from the TD children in 17 gait features. Additionally, 21 gait features evolved longitudinally when following-up the same boys with DMD and 25 gait features presented a significant cross-sectional baseline age-effect. The current study quantitatively described the longitudinal alterations in gait features in boys with DMD, thereby providing detailed insight into how DMD gait deteriorates. Additionally, our results highlight that gait features extracted from 3DGA are promising outcome measures for future clinical trials to quantify the efficacy of novel therapeutic strategies.

## Introduction

Duchenne muscular dystrophy (DMD) is an X-linked degenerative muscular disorder and is the most common muscular dystrophy in children, affecting one in 3,500–6,000 new-born boys (Sutherland et al., 1981; Emery and Eh, 1991; Sussman, 2002). DMD is caused by mutations in the gene encoding dystrophin (Sussman, 2002). Subsequently, the dystrophin protein, which is important for ensuring the stability of the muscle cell membrane and protecting the muscles from contraction-induced damage (Petrof et al., 1993; Moens et al., 1994; Lindsay et al., 2020), is deficient. Therefore, the muscles progressively degenerate as children with DMD get older (Sussman, 2002). This degeneration is characterized by progressive loss of contractile tissue, which is replaced by fat and fibrotic tissue (Sussman, 2002; Jones et al., 2010). The primary clinical symptom due to muscle damage is progressive muscle weakness (Sussman, 2002). Secondary symptoms include increasing muscle stiffness (i.e., increased resistance to muscle lengthening) and contractures (i.e., reduced range of motion) (Sussman, 2002; Bushby et al., 2010; Skalsky and McDonald, 2012). These muscle impairments contribute to a decline in motor function, such as rising from the floor and climbing stairs, eventually resulting in loss of ambulation (Sutherland et al., 1981; Sussman, 2002). To date, no curative treatment for DMD is available (Sussman, 2002). If untreated, these children become wheelchair-bound around the age of nine years and die at a mean age of 19 years due to cardiac or pulmonary failure (Bushby et al., 2005, 2010). However, multidisciplinary symptomatic medical, surgical, and rehabilitative treatment, including long-term use of corticosteroids, and ventilatory support, have altered the natural disease course and have increased life expectancy until approximately the age of 30 years (Bushby et al., 2010).

Even with the current state-of-the-art medical care, children with DMD lose ambulation at a mean age of 12 years (Ryder et al., 2017). Promising novel therapeutic strategies might alter the natural history of the disease such that the most affected children remain ambulant until 12 years of age (Yiu and Kornberg, 2015) and some children even remain ambulant over 15 years of age (Goemans et al., 2017). However, it has been difficult to transfer these new therapeutic interventions from clinical trials into clinical practice, as it has been very challenging to prove the efficacy of novel drugs in DMD (Goemans, 2013). Clinical trials often use the 6-min walk test (6MWT), the North Star Ambulatory Assessment (NSAA) and timed tests as evaluation tools (Mazzone et al., 2010; Goemans, 2013; Goudriaan et al., 2018a). These assessments reflect clinically meaningful aspects of daily life and have been proven reliable, valid, and feasible (Kierkegaard and Tollbäck, 2007; Frosini et al., 2009; McDonald et al., 2010). Therefore, clinicians have accepted these tools as standard clinical assessments to track the long-term progression of the disease and to predict loss of ambulation in children with DMD (McDonald et al., 1995; Mazzone et al., 2010). However, the 6MWT, the NSAA, and the timed tests measure global gross motor function and might not be sensitive enough to detect significant changes in the gait pattern of individuals with DMD. This might especially be true in the early disease stages, where compensation strategies are still successful (Kirschner et al., 2010; Lu et al., 2014; Lynn et al., 2015). Hence, there is an urgent need for sensitive, suitable, and clinically meaningful outcome measures to prove the successes of emergent therapeutic strategies (Goemans, 2013).

Three-dimensional gait analysis (3DGA) is proposed as an alternative evaluation method that can be used in clinical trials (Romano et al., 2019; Wren et al., 2020). It allows for a detailed analysis of gait pathology, including altered joint kinematics, kinetics and muscle activity patterns. The assessment of these parameters might be beneficial when evaluating the efficacy of novel therapeutic strategies (Heberer et al., 2016). While 3DGA is part of the standard clinical care in children with cerebral palsy (Bregou Bourgeois et al., 2014; Shin et al., 2016), this method is not implemented in the clinical follow-up of children with DMD (Bushby et al., 2010). Even though several gait features measured with 3DGA correlated strongly with clinical assessments, the correlation coefficients (ρ) ranged substantially, from -0.032 (range of ankle dorsiflexion/plantar flexion motion) to 0.858 (maximal dorsiflexion angle during swing) for the NSAA and from -0.022 (range of hip flexion/extension motion) to 0.850 (hip adduction/abduction mean angle) for the 6MWT (Romano et al., 2019). This stresses the added value of 3DGA to monitor the functional ability in children with DMD (Romano et al., 2019). Furthermore, previous studies indicated that 3DGA could provide detailed insight into disease progression and treatment response in children with DMD that may otherwise go undetected when only global function is studied (D’Angelo et al., 2009; Heberer et al., 2016). Lastly, to maintain a certain level of functionality and to postpone spinal deformities and muscle contractures, prolonging the ambulation is considered the most important treatment goal in children with DMD (Sussman, 2002). Measuring the progressive alterations in the gait pattern of boys with DMD in detail by means of 3DGA could provide sensitive outcome measures to evaluate the efficacy of novel therapeutic strategies (Heberer et al., 2016).

However, before 3DGA can be used as an assessment method in clinical trials, a full delineation of the progressive gait pathology in children with DMD is required. Goudriaan et al. (2018a) summarized previous studies that applied 3DGA to objectively quantify gait deviations in children with DMD (Goudriaan et al., 2018a). Due to insufficient description of the study population and measurement devices, small sample sizes, and wide age ranges contributing to heterogeneous groups, generalization of the findings is challenging. From the 79 gait parameters reported in literature, the studies only agreed on an increase in knee range of motion (D’Angelo et al., 2009; Doglio et al., 2011), a decrease in walking speed, stride length, step length, dorsiflexion angle in swing (Sutherland et al., 1981; D’Angelo et al., 2009; Gaudreault et al., 2010; Doglio et al., 2011), maximal knee extension moment (Khodadadeh et al., 1986; Gaudreault et al., 2010; Doglio et al., 2011), maximal dorsiflexion moment (Gaudreault et al., 2007, 2010; Doglio et al., 2011), maximal hip power generation (D’Angelo et al., 2009; Gaudreault et al., 2010), and maximal ankle power generation (D’Angelo et al., 2009; Gaudreault et al., 2010; Doglio et al., 2011) in the children with DMD when compared to typically developing (TD) children. Furthermore, even though DMD is a progressive disorder (Sussman, 2002), mostly cross-sectional studies including participants with a wide age range have been performed to investigate the differences in gait features between DMD and TD. While these studies give insight into the characteristics of DMD gait, they do not provide the complete picture of how their gait pattern deteriorates over time nor which gait features might be predictive for the loss of ambulation.

Sutherland et al. (1981) were one of the few to describe the progression of the gait pathology in children with DMD by introducing three stages, namely the early, transitional and late stage (Sutherland et al., 1981). A subtle effect of disease progression (e.g., muscle weakness) was already apparent in the early stage. This included slight backward leaning of the trunk with lumbar lordosis to compensate for weak hip extensors, and posterior tilt of the pelvis in combination with hip hyperextension. During swing, increased hip flexion probably compensated for the drop foot. Consequently, a flat foot strike at initial contact was seen in the young boys with DMD. Furthermore, an increased external foot progression was measured throughout the entire gait cycle. In the transitional stage, the early posterior tilt changed toward increased pelvic anterior tilt in combination with increased hip flexion, which represented the most remarkable characteristic. Sutherland et al. (1981) indicated this as a compensation mechanism for increased weakness in the knee extensors. Furthermore, boys with DMD walked with increased base of support, shoulder sway and lateral trunk leaning to compensate for weak hip abductors. In addition to the drop foot in swing, children in the transitional stage showed characteristics of equinus gait, namely forefoot strike at initial contact and reduced dorsiflexion in stance. Furthermore, a transition from external to internal foot progression occurred. Due to the progressive muscle weakness, the aforementioned compensation mechanisms became more pronounced in the late stage and therefore, the gait features in boys deviated even more from TD children (Sienko Thomas et al., 2010). The equinus gait at this late stage was also more evident, as the ankle joint was constantly in plantar flexion throughout the gait cycle (Sutherland et al., 1981). However, the classification of Sutherland et al. (1981) was introduced approximately 40 years ago (Sutherland et al., 1981). Meanwhile, the state-of-the-art medical care has altered the natural history of DMD, resulting in a longer ambulation period for boys with DMD (Sussman, 2002; Bushby et al., 2010). A later study from 2010 (Sienko Thomas et al., 2010), which classified the gait pattern in children with DMD based on the gait deviation index, could only partially confirm the stages of Sutherland et al. (1981). While these differences could to some extent be the result of different analysis methods, they emphasize a strong need for updated longitudinal 3DGA assessments in the same cohort of children with DMD.

The overall goal was to provide a full delineation of the progressive gait pathology in children with DMD, covering the entire period of ambulation, by performing a so-called mixed cross-sectional longitudinal study. To make our results comparable with previous literature, our first aim was to cross-sectionally compare 31 predefined gait features that were defined based on previous literature (Sutherland et al., 1981; Sienko Thomas et al., 2010; Goudriaan et al., 2018a) and case discussions in a clinical specialist team, between children with DMD and a TD database. We hypothesized that the gait features would be significantly different between the two groups, specifically when looking at the pelvis. The second aim was to explore the longitudinal changes in the 31 predefined gait features in growing boys with DMD using follow-up 3DGA sessions. We hypothesized that the gait features would change significantly over time as children with DMD grow older and the disease progresses.

## Materials and Methods

### Participants

Thirty children with DMD were recruited at the multidisciplinary clinic of the Neuro-Muscular Reference Centre (NMRC) in the University Hospitals Leuven. The following inclusion criteria were used: (1) diagnosed with DMD *via* immunohistochemistry, muscle biopsy and/or mutation of the dystrophin gene; (2) between three and 16 years old, (3) able to walk independently for at least 100 meters. Chronic treatment with corticosteroids and participation in clinical trials were permitted. Exclusion criteria were: cognitive and behavioral disorders preventing accurate measurements (1), history of lower limb surgery (2), clinical picture of Becker muscular dystrophy and genetic diagnosis predicting a milder phenotype, such as in-frame deletions (3). Twenty-seven TD boys of a similar age as the children with DMD, and without the presence of any neurological or neuromuscular disorder, were selected out of the reference database from the Clinical Motion Analysis Laboratory of the University Hospitals Leuven (Pellenberg).

All children were measured at the Clinical Motion Analysis Laboratory. This study was approved by the local ethics committee (Ethical Committee UZ Leuven/KU Leuven; S55867, S56041, and S61324) under the Declaration of Helsinki. Participants’ parents or caretakers signed a written informed consent after agreeing to participate in the study. Children of 12 years or older also signed an assent.

### Study Design

**FIGURE 1:**
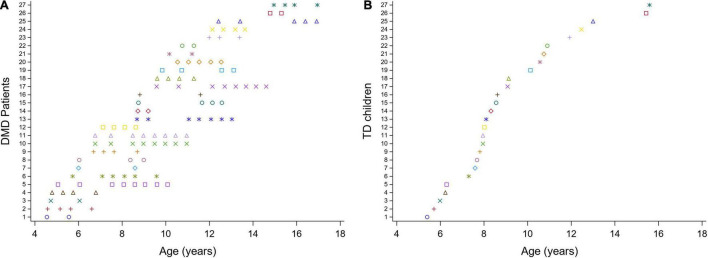
Mixed cross-sectional longitudinal dataset. Overview of the ages at the included measurements for the children with DMD **(A)** and for the TD database **(B)**. Each color represents one patient. DMD, Duchenne muscular dystrophy; TD, typically developing.

We performed a so-called mixed cross-sectional longitudinal study, with a cross-sectional component (children measured only once) and a longitudinal component (children with repeated measures over time) (Figure 1). The children with DMD enrolled in the study at different ages (range: 4.6–15 years) and were repeatedly measured over variable time intervals, establishing a longitudinal database. 3DGA’s were collected at multiple time points, with a frequency of two to nine times at a variable time interval of 5–35 months, covering a varying follow-up period ranging from 6 months to 5 years (average follow-up period: 2.5 years) in the boys with DMD. This resulted in an unbalanced dataset of 124 3DGA-sessions. The TD children (*n* = 27) were only measured at one time point, resulting in a cross-sectional database consisting of 27 3DGA sessions.

**FIGURE 2:**
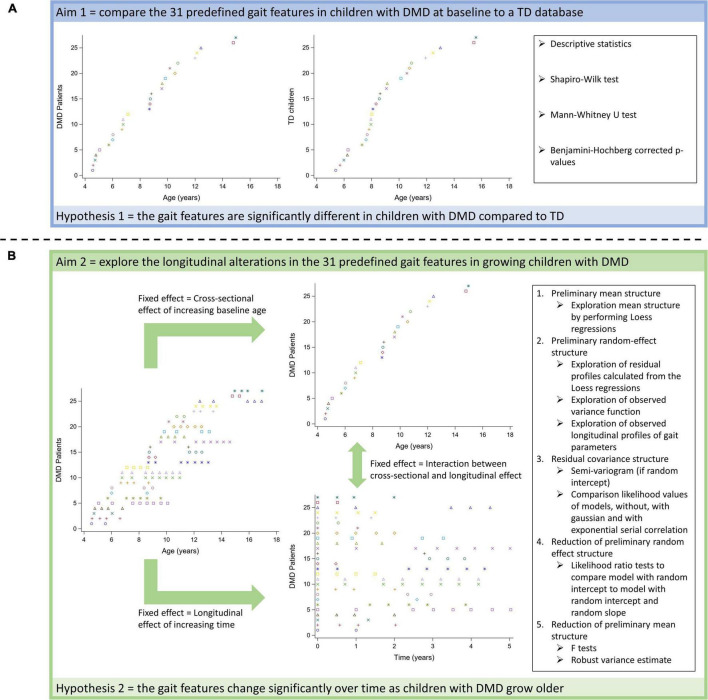
Schematic overview of statistical analyses to achieve the first **(A)** and the second aim **(B)**.

To achieve the first aim, we used a cross-sectional comparison to assess the differences in 31 predefined gait features between the children with DMD at the time of baseline assessment and the TD children (Figure 2A). To achieve the second aim, the changes in 31 predefined gait features in the same group of children with DMD were assessed from both a longitudinal as well as a cross-sectional perspective (Figure 2B). The time course of the measurements (time) represented the longitudinal effect. The effect of increasing age at the time of the first measurement (baseline age), represented the cross-sectional effect. This design was chosen, since the boys with DMD enrolled in the study at different ages resulting in the mixed longitudinal dataset.

### Data Collection

The 3DGA’s were conducted in barefoot condition, at self-selected walking speed on a 10-m walkway. Reflective markers with a diameter of 14 mm were attached to the skin according to the Plug-In Gait Full-Body model and a 10-15 Vicon camera system (Vicon-UK, Oxford, United Kingdom) sampled at 100 Hz recorded the marker trajectories. These trajectories were filtered with a built-in Woltring filter (mode: MSE; smoothing: 15 mm^2^). Two force plates (AMTI, Watertown, MA, United States) embedded in the walkway registered ground reaction forces at 1,500 Hz to calculate internal joint moments. Gait cycles were defined through manually indicating the gait events, with the support of force plate data if available, i.e., initial contacts and toe offs, in the Nexus software (Nexus 2.10. Vicon-UK, Oxford, United Kingdom). Subsequently, pelvis kinematic and lower limb joint kinematic and kinetic waveforms were estimated. For both sides, ten gait cycles with kinematic data, of which three to five gait cycles with kinetic data, were collected.

### Data Analysis

The quality of the collected gait cycles was checked with a custom-made software in MATLAB (The Mathworks Inc., Natick, M.A., 2016). Kinematic and kinetic waveforms were resampled to 101 data points per gait cycle. Kinematic waveforms were expressed in degrees. Kinetic waveforms were normalized to bodyweight and therefore, internal joint moments were expressed in Newton meters per kilogram bodyweight (Nm/kg), and power waveforms in Watt per kilogram bodyweight (W/kg). Per measurement session, the spatiotemporal parameters, and the kinematic and kinetic waveforms were averaged over the selected gait cycles with good quality for each side. Four spatiotemporal parameters were included: cadence (steps/s), walking velocity (WV), step length (SL), and step width (SW). Using the equations (Eq.) of Hof (Hof, 1996), WV, SL and SW were converted into non-dimensional quantities. WV was normalized to leg length and gravitational acceleration (Eq. 1). SL and SW were normalized to leg length (Eq. 2 and 3). Equations:


(1) $$W\,V_{n\,o\,r\,m}=\frac{W\,V}{\sqrt{l*g}}$$

(2) $$S\,L_{n\,o\,r\,m}=\frac{S\,L}{l}$$

(3) $$S\,W_{n\,o\,r\,m}=\frac{S\,W}{l}$$

with *WV*_*norm*_, *SL*_*norm*_, *SW*_*norm*_, *l*, and *g* representing normalized walking velocity, normalized step length, normalized step width, leg length, and gravitational acceleration, respectively.

From the average continuous kinematic and kinetic waveforms, minima, maxima, range of motions, and values at specific events in the gait cycle were calculated using the previously mentioned custom-made software, to obtain 16 predefined kinematic and 10 kinetic parameters. The TD database from the Clinical Motion Analysis Laboratory (*n* = 86) served as a reference to calculate one additional kinematic parameter, i.e., the gait profile score (GPS), for the DMD and TD group (Baker et al., 2009). The GPS is an overall gait index representing the difference in kinematics between an individual subject and a control group. In total, the dataset contained 31 predefined gait features (i.e., 4 spatiotemporal, 17 kinematic, and 10 kinetic parameters), which were selected based on previous literature (Sutherland et al., 1981; Sienko Thomas et al., 2010; Goudriaan et al., 2018a) and clinical expertise.

Since children with DMD may present an asymmetric gait pattern, gait features of the right and the left side were not averaged. To avoid a complex dataset with the inclusion of both sides on each time point per patient, only the gait features of the weakest side for the children with DMD were used in further analyses. This side was determined based on the strength outcomes of the clinical exam (manual muscle testing). For the TD children and in case no weakest side could be determined for the children with DMD, a random number generator was used to define the evaluated side.

### Statistical Analysis

An overview of all statistical analyses per research aim is provided in Figure 2.

We used descriptive statistics to provide an overview of the studied gait features in both groups (DMD and TD) for the first aim (Figure 2A). Depending on the distribution of the data, means and standard deviations or medians and interquartile ranges were reported.

To verify our hypotheses related to the first aim, we first checked the normality of the data by inspecting the distribution plots and *via* the Shapiro-Wilk test. Since most of the parameters were not normally distributed, a Mann-Whitney *U* test was performed to investigate differences in subject characteristics and gait features between the children with DMD at baseline and the TD children. The *p*-values were corrected according to the stepwise Benjamini-Hochberg procedure with a false discovery rate of 5% to correct for the comparison of 36 parameters (Benjamini and Hochberg, 1995).

To investigate the longitudinal evolution of the pathological gait pattern in the children with DMD (second aim; Figure 2B), linear mixed effect models (LMM) were used. These methods were required to handle the unbalanced mixed longitudinal dataset, as the number of repeated measurements and the time interval between measurements varied among the children with DMD (Verbeke and Molenberghs, 2000). LMM take the correlation between repeated measurements into account by modeling the variance both between and within the patients. Therefore, fitting LMM requires specification of a mean structure and a covariance structure (Verbeke and Molenberghs, 1997, 2000). The mean structure consists of fixed effects, i.e., time effects, covariates, and interaction effects, while the covariance structure consists of random effects and serial correlation (Verbeke and Molenberghs, 2000). The time course of the measurements within the patients (time), which represented the longitudinal effect was selected as the time effect (Eq. 4). In case of a quadratic relationship between time and the gait feature, the higher-order term of time, i.e., time squared (time^2^), was added in the LMM. Baseline age, which represented the cross-sectional effect of increasing age was selected as a covariate (Eq. 4). The higher-order terms of baseline age, i.e., baseline age squared (baseline age^2^) and baseline age cubed (baseline age^3^), were added in the LMM, when a quadratic and cubic relationship between baseline age and the gait feature were observed, respectively. In addition, the longitudinal effect may depend on the baseline age. Therefore, the interaction between time and baseline age (time × baseline age) was also included as a fixed effect in the LMM (Eq. 4). To model the variability among the boys with DMD, random effects consisting of a random intercept and a random slope for the time effect, were added (Eq. 4). A random intercept and slope account for the variability in starting point and progression rate between patients, respectively (Verbeke and Molenberghs, 2000). To model the variability within the participants, measurement error, and serial correlation were added in the LMM (Eq. 4). Serial correlation is needed when the error term is not fully described by the constant variance of the measurement error and there is still, after adding random effects, a certain correlation between serial measurements (Verbeke and Molenberghs, 2000). This correlation usually decreases in function of the time separation between the repeated measurements. Serial correlation represents the belief that part of an individual’s observed profile is a response to time-varying stochastic processes operating within that patient. The gait features were defined as the responses.

$$R\,e\,s\,p\,o\,n\,s\,e\,s=\beta_{0}+\beta_{1}\times T\,i\,m\,e+\beta_{2}\times T\,i\,m\,e^{2}+\beta_{3}\times B\,a\,s\,e\,l\,i\,n\,e\,a\,g\,e+\beta_{4}$$

$$\times B\,a\,s\,e\,l\,i\,n\,e\,a\,g\,e^{2}+\beta_{5}\times B\,a\,s\,e\,l\,i\,n\,e\,a\,g\,e^{3}+\beta_{6}\times T\,i\,m\,e$$

(4) $$\times B\,a\,s\,e\,l\,i\,n\,e\,a\,g\,e+b_{1\,i}+b_{2\,i}\times T\,i\,m\,e+\varepsilon_{\left(1\right)\,i\,j}+\varepsilon_{\left(2\right)\,i\,j}$$

with β_0_, β_1_, β_2_, β_3_, β_4_, β_5_, β_6_, *b*_*1i*_, *b*_*2i*_, ε_(1)*ij*_, ε_(2)*ij*_, representing intercept, regression coefficient for Time, regression coefficient for Time^2^, regression coefficient for Baseline age, regression coefficient for Baseline age^2^, regression coefficient for Baseline age^3^, regression coefficient for the interaction between Time and Baseline age, random intercept, random slope for Time, measurement error, and serial correlation, respectively. Both time and baseline age were expressed in years. From baseline age the smallest value was subtracted. Therefore, the intercept represents the response’s value at baseline age 4.6 and time zero.

We used the following workflow to construct the LMM (Figure 2B; Verbeke and Molenberghs, 1997, 2000). First, the average trend was explored by performing Loess regressions (point 1, Figure 2B). Based on visual inspection of the smoothed line resulting from the Loess regression, the higher-order terms of the fixed effects were selected to build a comprehensive preliminary mean structure (Verbeke and Molenberghs, 2000; Diggle et al., 2002). Second, the preliminary random-effect structure was defined based on the exploration of the residual profiles calculated from the Loess regressions and the observed variance function, i.e., the change of the squared residuals over time (point 2, Figure 2B). In addition, it was informally checked if the observed longitudinal profiles could be well-explained by a specific linear regression function with a SAS ^®^ macro (metagof.sas online available at https://ibiostat.be/online-resources) (Verbeke and Molenberghs, 1997). Based on the data exploration, a random-effect structure with solely a random intercept, i.e., assuming a constant variance, or a random intercept and slope, i.e., allowing the variance to change over time, was selected. Third, the residual covariance structure was selected (point 3, Figure 2B). The semi-variogram (Diggle, 1988) was used to explore the presence and the nature of serial correlation only when a random intercept was included. The random intercept captures child-specific characteristics, whereas serial correlation accounts for the fact that measurements closer to each other in time are typically more strongly correlated than measurements farther apart within the measurement sequence of a given child. The likelihood values of three models with the preliminary mean and random-effect structures, one without, one with Gaussian, and one with exponential serial correlation were compared to define the residual covariance structure. Precisely, they are based on so-called restricted maximum likelihood, a modification of classical maximum likelihood to alleviate small-sample biases. Fourth, it was checked if the number of random effects in the preliminary random-effects structure could be reduced by performing the likelihood ratio test to compare a model with a random intercept vs. a model with a random intercept and slope (point 4, Figure 2B). For technical reasons, i.e., because the variance components need to form a positive-definite matrix, these likelihood ratios follow a mixture of χ^2^ distributions (χ^2^_1:2_) needed to be used to detect significance in the results. Fifth, the reduction of the number of fixed effects was checked by performing F tests (point 5, Figure 2B). To correct for possible misspecification of the covariance matrix, the robust variance estimate, also called sandwich estimate, was calculated. This way, robust inference of the F test could be obtained. Due to the explorative nature of the second aim, the *p*-values were not corrected for multiple comparisons.

After formulating the LMM, so-called empirical Bayes estimates were calculated and used to detect outliers, i.e., patients with an “exceptional” starting point and evolution over time. The data of the outliers were checked. If data were removed, the previous five steps were repeated to define the final LMM.

All statistical analyses were performed using SAS ^®^, version 9.4 (Statistical Analysis Software 9.4, SAS Institute Inc., Cary, NC, United States).

## Results

In total, 27 boys with DMD aged between 4.6 and 15 years old at baseline [median age (interquartile range): 8.7 (4.5) years] and repeatedly measured (range: two to nine measurements) over a varying follow-up period (range: 6 months–5 years) were included in the study. The control group consisted of 27 TD children aged between 5.4 and 15.6 years old [median age (interquartile range): 8.3 (3.1) years] (Table 1).

**TABLE 1:** Subject characteristics of boys with DMD at baseline and TD children, and results from MWU-test.

	Median (Q1-Q3)		MWU-test
Variables	DMD	TD	BH corrected *p*-value
Gender	Boys: 27	Boys: 27	
Age (years)	8.7 (6.0–10.5)	8.3 (7.6–10.7)	0.5500
Weight (kg)	23.7 (19.7–36.0)	27.2 (23.3–37.0)	0.2437
Height (m)	1.21 (1.08–1.31)	1.33 (1.24–1.49)	0.0033*
BMI (kg/m^2^)	17.6 (16.3–21.9)	15.6 (15.0–17.8)	0.0070*
Leg length (m)	0.58 (0.50–0.65)	0.65 (0.61–0.78)	0.0024*

The asterisks (*) indicate significance level at BH corrected p-value < 0.05. BH, Benjamini-Hochberg procedure; BMI, Body Mass Index; DMD, Duchenne muscular dystrophy; MWU, Mann-Whitney U; TD, typically developing.

All the collected 3DGA sessions of three patients and only one follow-up session for four patients were excluded due to obvious marker misplacement, inconsistent walking and/or behavioral disturbances leading to inaccurate measurements. The measurements with marker misplacement (*n* = 2) were only included for the spatiotemporal parameters, but excluded for the kinematic and kinetic parameters. This resulted in a final dataset of 115 and 113 measurements for the spatiotemporal and kinematic/kinetic data, respectively.

### Aim 1: Gait Features in Children With Duchenne Muscular Dystrophy at Baseline Compared to Typically Developing Children

#### Spatiotemporal Parameters

At baseline, the boys with DMD presented a lower WV_norm_ (*p* = 0.0036) and smaller SL_norm_ (*p* = 0.0281) compared to TD (Table 2). The SW_norm_ was significantly broader in DMD compared to TD (*p* = 0.0036).

**TABLE 2:** Group differences in gait features between DMD at baseline and TD, and results from MWU-test.

	Median (Q1-Q3)		MWU-test
Variables	DMD	TD	BH corrected *p*-value
Cadence (steps/s)	2.25 (2.01–2.40)	2.21 (1.97–2.47)	0.4639
WVnorm (/)	0.40 (0.37–0.43)	0.46 (0.43–0.51)	0.0036*
SWnorm (/)	0.28 (0.24–0.30)	0.16 (0.11–0.19)	0.0036*
SLnorm (/)	0.74 (0.70–0.79)	0.77 (0.74–0.85)	0.0281*
Gait profile score (°)	6.81 (5.13–8.37)	5.21 (4.87–6.57)	0.0924
Max anterior pelvic tilt (°)	16.36 (14.30–19.03)	11.64 (8.69–15.26)	0.0047*
ROM pelvic obliquity (°)	9.62 (7.50–13.32)	7.42 (6.97–9.20)	0.0502
ROM pelvic rotation (°)	14.41 (9.96–17.18)	14.93 (11.21–17.49)	0.7535
Min hip flex angle stance (°)	−3.08 (−7.68 to 3.77)	−10.13 (−13.32 to −6.84)	0.0006*
Max hip flex angle swing (°)	41.81 (34.67–49.01)	34.12 (29.19–37.89)	0.0018*
ROM hip sagittal plane (°)	42.70 (38.90–46.95)	45.42 (41.02–47.77)	0.2578
Max hip ext mom stance (Nm/kg)	0.507 (0.388–0.688)	1.098 (0.699–1.319)	0.0012*
Min hip ext mom stance (Nm/kg)	−0.698 (−0.789 to −0.516)	−0.904 (−1.068 to −0.772)	0.0009*
Max hip power stance (W/kg)	0.440 (0.334–0.775)	0.665 (0.445–1.094)	0.0554
Min hip add angle stance (°)	−6.79 (−8.36 to −3.96)	−4.92 (−6.29 to −2.92)	0.0501
Min hip add angle swing (°)	−9.45 (−11.38 to −8.03)	−6.50 (−7.14 to −5.34)	0.0018*
Max hip abd mom stance (Nm/kg)	0.559 (0.443–0.735)	0.648 (0.581–0.723)	0.0582
Max knee flex angle stance (°)	35.02 (33.16–39.36)	35.55 (28.03–40.28)	0.7722
Min knee flex angle stance (°)	9.06 (3.86–12.31)	3.65 (−2.54–7.62)	0.0070*
ROM knee sagittal plane stance (°)	28.01 (24.31–30.70)	30.72 (28.06–33.23)	0.0070*
Max knee flex angle swing (°)	69.48 (64.38–74.45)	64.75 (60.05–67.84)	0.0038*
Max knee ext mom stance (Nm/kg)	0.414 (0.337–0.490)	0.512 (0.325–0.663)	0.1263
Min knee ext mom stance (Nm/kg)	−0.049 (−0.120 to 0.022)	−0.207 (−0.334 to −0.157)	0.0007*
Dorsiflex angle IC (°)	0.80 (−3.61 to 4.07)	0.77 (−1.70 to 3.77)	0.9586
Max dorsiflex angle stance (°)	15.09 (11.34–17.20)	12.53 (9.47–16.55)	0.3147
Max dorsiflex angle swing (°)	5.31 (0.02–6.95)	5.27 (2.56–8.35)	0.4202
Min plantar flex mom LR (Nm/kg)	−0.050 (−0.088 to −0.008)	−0.127 (−0.184 to −0.069)	0.0012*
Max plantar flex mom PO (Nm/kg)	0.976 (0.864–1.185)	1.318 (1.141–1.446)	0.0009*
Min ankle power LR (W/kg)	−0.501 (−0.941 to −0.265)	−0.467 (−0.605 to −0.349)	0.9128
Max ankle power PO (W/kg)	3.022 (2.271–3.384)	3.973 (3.100–4.386)	0.0020*
Max int foot prog angle stance (°)	−7.96 (−11.96 to −3.13)	0.11 (−4.16 to 4.22)	0.0005*

The asterisks (*) indicate significance level at BH corrected p-value < 0.05. abd, abduction; add, adduction; BH, Benjamini-Hochberg procedure; DMD, Duchenne muscular dystrophy; dorsiflex, dorsiflexion; ext, extension; flex, flexion; IC, initial contact; int, internal; LR, loading response; Max, maximal; Min, minimal; mom, moment; MWU, Mann-Whitney U; PO, push-off; prog, progression; ROM, range of motion; SLnorm, normalized step length; SWnorm, normalized step width; TD, typically developing; WVnorm, normalized walking velocity.

#### Kinematic and Kinetic Parameters

The maximal anterior pelvic tilt angle was larger in DMD compared to TD (*p* = 0.0047) (Table 2). The minimal and maximal hip flexion angles during stance (*p* = 0.0006) and swing (*p* = 0.0018), respectively, were larger in the children with DMD compared to TD. The maximal hip extension moment was smaller (*p* = 0.0012), while the minimal hip extension moment was larger (*p* = 0.0009) in DMD compared to TD. The boys with DMD presented a smaller minimal hip adduction angle during swing (*p* = 0.0018) than TD children. The boys with DMD walked with a larger minimal knee flexion angle during stance than TD children (*p* = 0.0070). The range of knee flexion/extension motion during the stance phase was smaller in DMD compared to TD (*p* = 0.0070). Boys with DMD walked with a larger maximal knee flexion angle during swing than TD children (*p* = 0.0038). The minimal knee extension moment was larger in DMD compared to TD (*p* = 0.0007). Minimal plantar flexion moment during loading response was larger (*p* = 0.0012), while the maximal plantar flexion moment (*p* = 0.0009) and ankle power (*p* = 0.0020) during push-off were smaller in the children with DMD compared to TD. Lastly, boys with DMD walked with a smaller maximal internal foot progression angle than TD children (*p* = 0.0005).

### Aim 2: Longitudinal Evolution of Gait Features in Growing Boys With Duchenne Muscular Dystrophy

**FIGURE 3:**
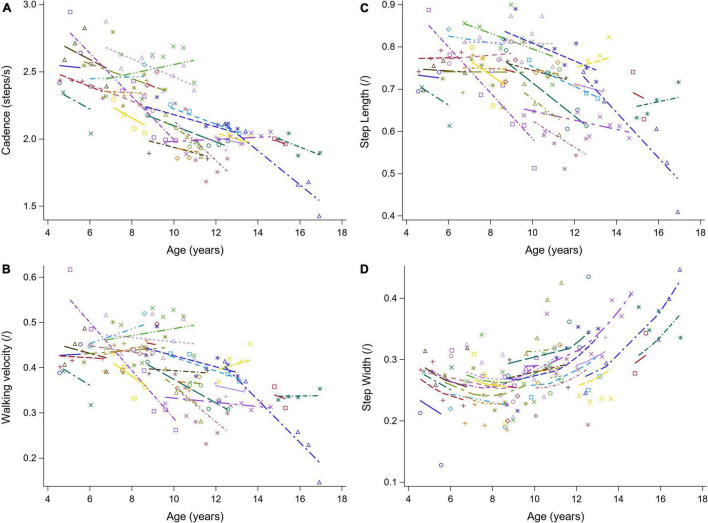
The individual predicted profiles (dashed lines) for cadence **(A)**, normalized walking velocity **(B)**, normalized step length **(C)**, and normalized step width **(D)**. The actual observed values are visualized by the symbols. Each color represents one patient with DMD. The regression coefficients of the fixed effects are given in Table 3. DMD, Duchenne muscular dystrophy.

**FIGURE 4:**
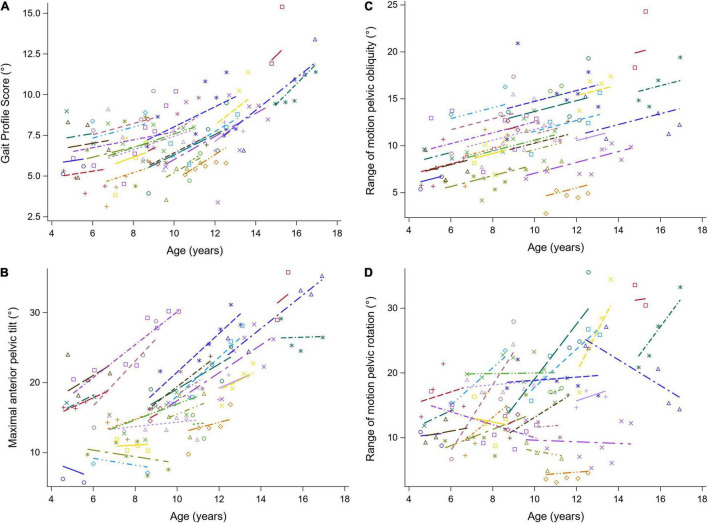
The individual predicted profiles (dashed lines) for the GPS **(A)**, the maximal anterior pelvic tilt **(B)**, range of motion pelvic obliquity **(C)**, and range of motion pelvic rotation **(D)**. The actual observed values are visualized by the symbols. Each color represents one patient with DMD. The regression coefficients of the fixed effects are given in Table 4. DMD, Duchenne muscular dystrophy; GPS, Gait Profile Score.

**FIGURE 5:**
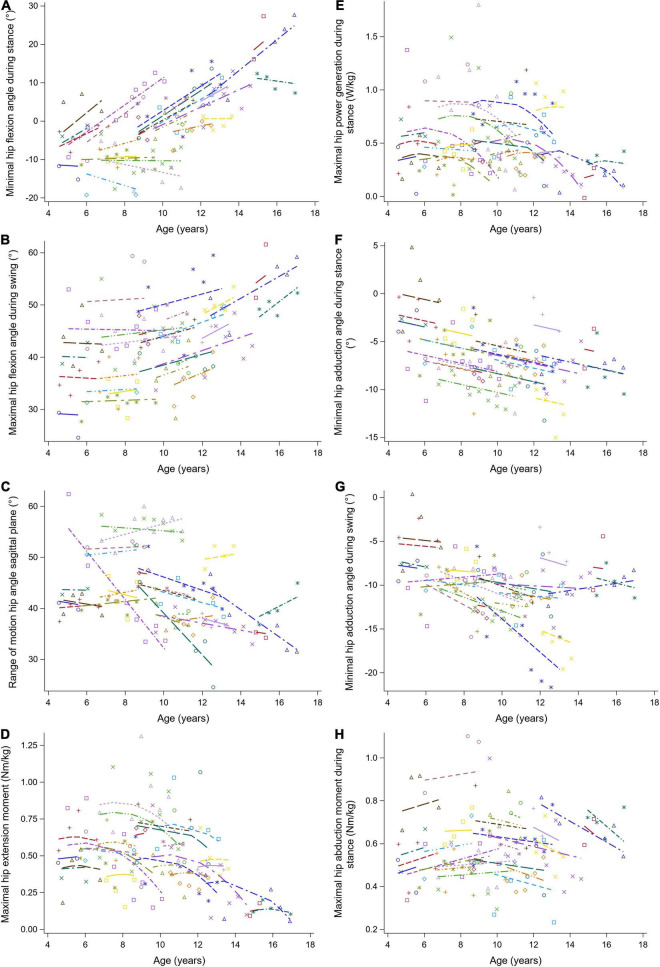
The individual predicted profiles (dashed lines) for the minimal hip flexion angle during stance **(A)**, maximal hip flexion angle during swing **(B)**, range of hip motion in the sagittal plane **(C)**, maximal hip extension moment **(D)**, maximal hip power generation during stance **(E)**, minimal hip adduction angle during stance **(F)**, minimal hip adduction angle during swing **(G)**, and maximal hip abduction moment during stance **(H)**. The actual observed values are visualized by the symbols. Each color represents one patient with DMD. The regression coefficients of the fixed effects are given in Table 5. DMD, Duchenne muscular dystrophy.

**FIGURE 6:**
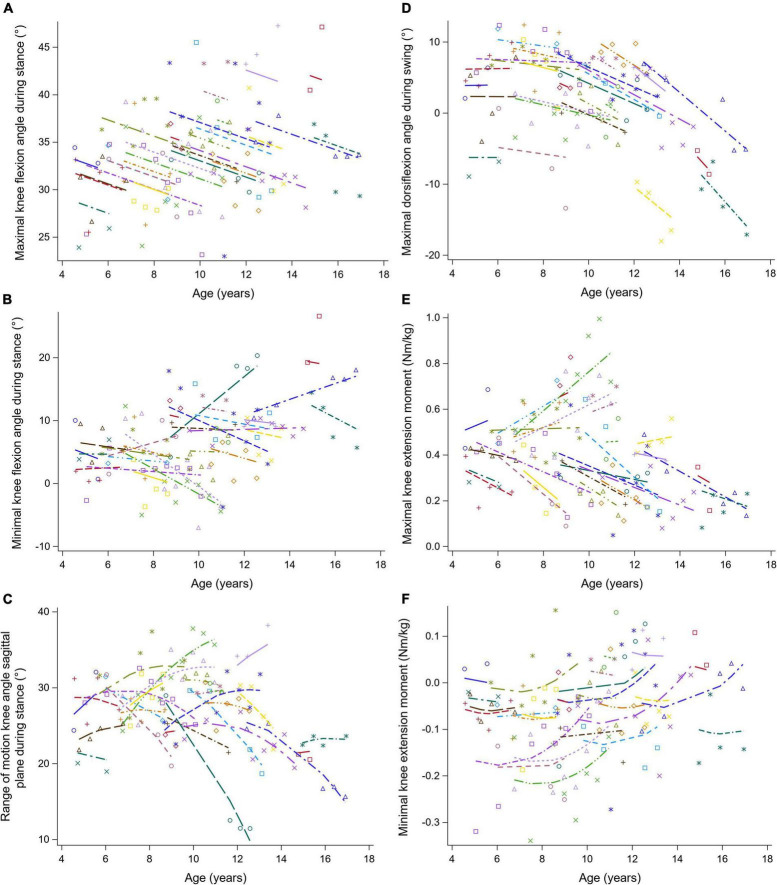
The individual predicted profiles (dashed lines) for the maximal knee flexion angle during stance **(A)**, minimal knee flexion angle during stance **(B)**, range of knee motion in sagittal plane during stance **(C)**, maximal knee flexion angle during swing **(D)**, maximal knee extension moment **(E)**, and minimal knee extension moment **(F)**. The actual observed values are visualized by the symbols. Each color represents one patient with DMD. The regression coefficients of the fixed effects are given in Table 6. DMD, Duchenne muscular dystrophy.

**FIGURE 7:**
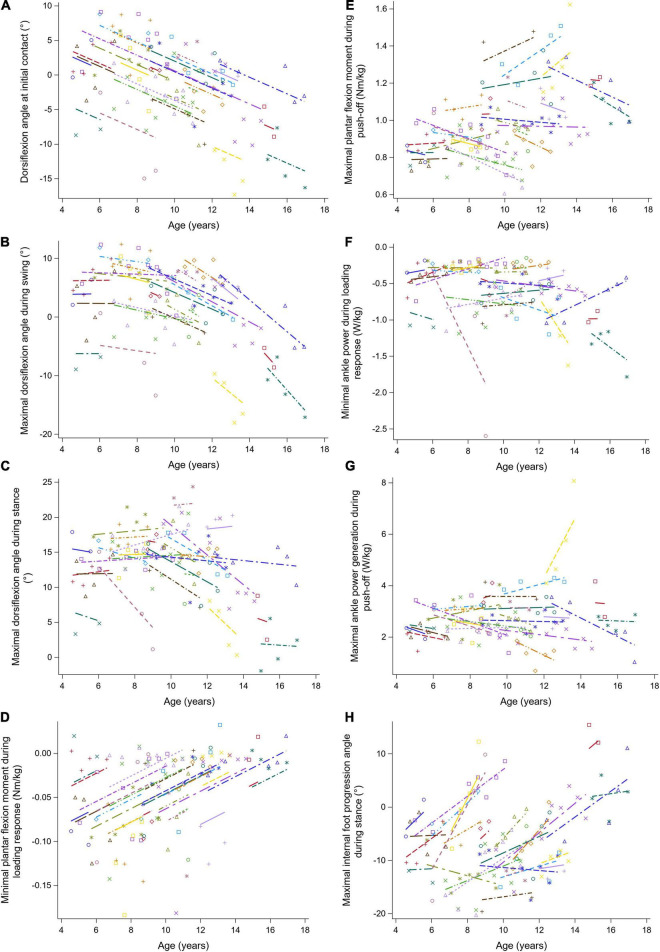
The individual predicted profiles (dashed lines) for the dorsiflexion angle at initial contact **(A)**, maximal dorsiflexion angle during swing **(B)**, maximal dorsiflexion angle during stance **(C)**, minimal plantar flexion moment during loading response **(D)**, maximal plantar flexion moment during push-off **(E)**, minimal ankle power during loading response **(F)**, maximal ankle power generation during push-off **(G)**, and maximal internal foot progression angle during stance **(H)**. The actual observed values are visualized by the symbols. Each color represents one patient with DMD. The regression coefficients of the fixed effects are given in Table 7. DMD, Duchenne muscular dystrophy.

The results of the LMM are presented in Tables 3–7. Per gait feature (i.e., each row in a table), the estimates of the fixed effects with the corresponding *p*-values are presented. For example, in Table 3, for the outcome SW_norm_, 0.262 (*p* < 0.05), -0.027 (*p* = 0.0006), 0.005 (*p* = 0.0016), 0.003 (*p* = 0.3895), 0.005 (*p* < 0.0001) are the estimates of the intercept, the regression coefficient of the linear time effect, the regression coefficient of the quadratic time effect, the regression coefficient of the baseline age effect, and the regression coefficient of the interaction effect between time and baseline age, respectively. The 95% confidence interval of the fixed effects’ estimates, and the random-effect and residual covariance structures are presented in Supplementary Tables 1, 2, respectively. Additionally, the individual predicted profiles are visualized in Figures 3–7.

**TABLE 3:** Results of linear mixed effect models with spatiotemporal parameters as responses in children with DMD.

	Fixed effects										
	Intercept	Regression coefficients									
		Longitudinal effects				Cross-sectional effects				Interaction effects	
		Time		Time^2^		Baseline age		Baseline age^2^		Time × Baseline age	
Variables	β_0_	β_1_	*p*-value	β_2_	*p*-value	β_3_	*p*-value	β_4_	*p*-value	β_6_	*p*-value
Cadence (steps/s)	2.617*	−0.058	0.0019*			−0.124	0.0001*	0.006	0.0266*		
WVnorm (/)	0.457*	−0.012	0.0613			−0.011	<0.0001*				
SLnorm (/)	0.785*	−0.016	0.0214*			−0.009	0.0026*				
SWnorm (/)	0.262*	−0.027	0.0006*	0.005	0.0016*	0.003	0.3895			0.005	<0.0001*

The asterisks (*) indicate significance level at p < 0.05. DMD, Duchenne muscular dystrophy; SLnorm, normalized step length; SWnorm, normalized step width; WVnorm, normalized walking velocity.

**TABLE 4:** Results of linear mixed effect models with the gait profile score and pelvis kinematics as responses in children with DMD.

	Fixed effects								
	Intercept	Regression coefficients							
		Longitudinal effects		Cross-sectional effects				Interaction effects	
		Time		Baseline age		Baseline age^2^		Time × Baseline age	
Variables	β_0_	β_1_	*p*-value	β_3_	*p*-value	β_4_	*p*-value	β_6_	*p*-value
Gait profile score (°)	6.69*	0.19	0.4649	−0.57	0.0797	0.09	0.0134*	0.10	0.0362*
Max anterior pelvic tilt (°)	15.71*	1.33	0.0004*	−1.42	0.0853	0.26	0.0003*		
ROM pelvic obliquity (°)	7.60*	0.58	0.0012*	0.74	0.0004*				
ROM pelvic rotation (°)	13.32*	1.35	0.0296*	−1.27	0.1577	0.25	0.0067*		

The asterisks (*) indicate significance level at p < 0.05. DMD, Duchenne muscular dystrophy; Max, maximal; ROM, range of motion.

**TABLE 5:** Results of linear mixed effect models with the hip kinematics and kinetics as responses in children with DMD.

	Fixed effects										
	Intercept	Regression coefficients									
		Longitudinal effects		Cross-sectional effects						Interaction effects	
		Time		Time^2^		Baseline age		Baseline age^2^		Time*Baseline age	
Variables	β_0_	β_1_	*p*-value	β_2_	*p*-value	β_3_	*p*-value	β_4_	*p*-value	β_6_	*p*-value
Min hip flex angle stance (°)	−7.63*	1.88	0.0022*			−0.22	0.8131	0.23	0.0133*		
Max hip flex angle swing (°)	37.10*	−0.22	0.7167			1.08	0.0075*			0.30	0.0049*
ROM hip sagittal plane (°)	47.10*	−0.60	0.2100			−0.76	0.0267*				
Max hip ext mom stance (Nm/kg)	0.502*	0.035	0.3421	−0.020	0.0104*	0.065	0.0141*	−0.010	< 0.0001*		
Max hip power stance (W/kg)	0.475*	0.066	0.3015	−0.030	0.0484*	0.061	0.0927	−0.008	0.0166*		
Min hip add angle stance (°)	−3.32*	−0.42	0.0250*			−1.24	0.0026*	0.094	0.0110*		
Min hip add angle swing (°)	−6.92*	−0.48	0.0930			−1.42	0.0013*	0.12	0.0009*		
Max hip abd mom stance (Nm/kg)	0.537*	0.029	0.0166*			0.017	0.0238*			−0.010	0.0024*

The asterisks (*) indicate significance level at p < 0.05. abd, abduction; add, adduction; DMD, Duchenne muscular dystrophy; ext, extension; flex, flexion; Max, maximal; Min, minimal; mom, moment; ROM, range of motion.

**TABLE 6:** Results of linear mixed effect models with knee kinematics and kinetics as responses in children with DMD.

	Fixed effects										
	Intercept	Regression coefficients									
		Longitudinal effects				Cross-sectional effects					
		Time		Time^2^		Baseline age		Baseline age^2^		Baseline age^3^	
Variables	β_0_	β_1_	*p*-value	β_2_	*p*-value	β_3_	*p*-value	β_4_	*p*-value	β_5_	*p*-value
Max knee flex angle stance (°)	32.14*	−0.85	0.0010*			0.73	0.0094*				
Min knee flex angle stance (°)	3.73*	−0.49	0.3203			1.00	0.0006*				
ROM knee sagittal plane stance (°)	26.06*	0.61	0.3377	−0.36	0.0033*	1.03	0.1231	−0.12	0.0361*		
Max knee flex angle swing (°)	68.83*	−1.25	0.0029*								
Max knee ext mom stance (Nm/kg)	0.418*	−0.018	0.3034								
Min knee ext mom stance (Nm/kg)	−0.043	−0.017	0.2387	0.008	0.0111*	−0.068	0.0302*	0.020	0.0176*	−0.001	0.0229*

The asterisks (*) indicate significance level at p < 0.05. DMD, Duchenne muscular dystrophy; ext, extension; flex, flexion; Max, maximal; Min, minimal; mom, moment; ROM, range of motion.

**TABLE 7:** Results of linear mixed effect models with ankle kinematics and kinetics as responses in children with DMD.

	Fixed effects								
	Intercept	Regression coefficients							
		Longitudinal effects		Cross-sectional effects				Interaction effects	
		Time		Baseline age		Baseline age^2^		Time × Baseline age	
Variables	β_0_	β_1_	*p*-value	β_3_	*p*-value	β_4_	*p*-value	β_6_	*p*-value
Dorsiflex angle IC (°)	1.70	−1.20	<0.0001*	0.52	0.5141	−0.15	0.0338*		
Max dorsiflex angle stance (°)	11.34*	−0.59	0.1390	2.64	0.0010*	−0.32	<0.0001*		
Max dorsiflex angle swing (°)	2.14	0.06	0.9074	2.09	0.0202*	−0.29	<0.0001*	−0.35	0.0003*
Min plantar flex mom LR (Nm/kg)	−0.060*	0.010	<0.0001*						
Max plantar flex mom PO (Nm/kg)	0.867*	−0.009	0.4905	0.037	<0.0001*				
Min ankle power LR (W/kg)	−0.533*	−0.026	0.5465	0.059	0.2426	−0.011	0.0196*		
Max ankle power PO (W/kg)	2.875*	−0.026	0.7813						
Max int foot prog angle stance (°)	−6.29*	2.10	0.0019*	−3.06	0.0021*	0.40	0.0006*		

The asterisks (*) indicate significance level at p < 0.05. DMD, Duchenne muscular dystrophy; dorsiflex, dorsiflexion; flex, flexion; IC, initial contact; int, internal; LR, loading response; Max, maximal; Min, minimal; mom, moment; PO, push-off; prog, progression; ROM, range of motion.

#### Spatiotemporal Parameters

The results of the LMM for the spatiotemporal parameters are given in Table 3. A longitudinal decrease in cadence of 0.058 steps/s per year was observed (Figure 3A). In addition, cadence decreased with baseline age, with a larger decrease before the age of nine and a smaller decrease after the age of nine. Only a small baseline age-effect was detected for WV_norm_, resulting in a decrease of 0.011 per year (Figure 3B). SL_norm_ decreased significantly longitudinally and with baseline age, with 0.016 and 0.009 per year, respectively (Figure 3C). For SW_norm,_ the longitudinal change was related to baseline age, i.e., a slow decrease and a rapid increase were detected before and after the baseline age of nine, respectively (Figure 3D).

#### Kinematic and Kinetic Parameters

The results of the LMM for the GPS and the pelvis kinematics are given in Table 4. The longitudinal change in GPS was related to age, i.e., the GPS increased more rapidly with increasing age (Figure 4A). A baseline age-effect resulted in a small decrease in GPS before the age of nine and a large increase in GPS after the age of nine. A longitudinal increase of 1.33 degrees per year in the maximal anterior pelvic tilt was found (Figure 4B). In addition, a small baseline age-effect resulted in a decreasing maximal anterior pelvic tilt before the age of nine, while above the age of nine, a larger baseline age-effect resulted in an increasing maximal anterior pelvic tilt. Furthermore, the pelvis range of motion (i.e., obliquity and rotation) increased longitudinally and with baseline age (Figures 4C,D). For pelvic obliquity, the range of motion increased 0.58 and 0.74 degrees, longitudinally and with baseline age, respectively. The pelvis rotation range of motion increased longitudinally with 1.35 degrees per year. With increasing baseline age, the pelvis rotation range of motion decreased until the age of seven and increased after the age of nine.

The results of the LMM for the hip kinematics and kinetics are given in Table 5. A longitudinal increase in the minimal hip flexion angle during stance of 1.88 degrees per year was found (Figure 5A). In addition, this angle increased progressively with baseline age. The longitudinal increase in the maximal hip flexion angle during swing was related to increasing baseline age, i.e., this angle increased more rapidly with baseline age (Figure 5B). The maximal hip flexion angle during swing also increased with baseline age. The range of hip flexion/extension motion decreased with baseline age (Figure 5C). Similar patterns, i.e., an increase followed by a decrease, were detected longitudinally for the maximal hip extension moment and the maximal hip power (Figures 5D,E). In addition, before the age of nine, the maximal hip extension moment and the maximal hip power increased with baseline age, while after the age of nine these gait features decreased. A longitudinal decrease in the minimal hip adduction angle during stance was detected (Figure 5F). Before the age of ten, the minimal hip adduction angles during stance and swing (Figure 5G) decreased with baseline age, while these gait features increased above the age of ten. The longitudinal change in the maximal hip abduction moment was related to baseline age, i.e., a longitudinal increase and decrease were detected before and after the baseline age of nine, respectively (Figure 5H). Additionally, the maximal hip abduction moment increased with baseline age.

The results of the LMM for the knee kinematics and kinetics are given in Table 6. The maximal knee flexion angle during stance decreased longitudinally with 0.85 degrees per year (Figure 6A). With increasing baseline age, the maximal and minimal knee flexion angles during stance increased with 0.73 and 1.00 degrees per year, respectively (Figures 6A,B). A longitudinal pattern, i.e., an increase followed by a decrease, was visible in the range of knee flexion/extension motion (Figure 6C). Before and after the age of nine, the range of knee flexion/extension motion increased and decreased with baseline age, respectively. The maximal knee flexion angle during swing decreased longitudinally with 1.25 degrees per year (Figure 6D). Longitudinally, a slow decrease followed by a rapid increase in the minimal knee extension moment was observed (Figure 6F). In contrast, the minimal knee extension moment showed an alternating pattern with increasing baseline age, i.e., firstly a decrease, secondly an increase, lastly another decrease.

The results of the LMM for the ankle kinematics and kinetics are given in Table 7. A longitudinal decrease of 1.20 degrees in the dorsiflexion angle at initial contact per year was found (Figure 7A). Additionally, this angle decreased progressively with baseline age. During swing, the longitudinal change in the maximal dorsiflexion angle was related to age, i.e., this angle decreased more rapidly with baseline age (Figure 7B). Before the age of nine, the maximal dorsiflexion angle in stance and swing increased, while after the age of nine, these gait features decreased with baseline age (Figures 7B,C). The minimal plantar flexion moment during loading response increased longitudinally with 0.010 Nm/kg per year (Figure 7D). The increasing baseline age resulted in an increase of 0.037 Nm/kg per year in the maximal plantar flexion moment during push-off (Figure 7E). The minimal ankle power during loading response decreased progressively with baseline age (Figure 7F). Longitudinally, a progression toward an internal foot progression angle was visible (Figure 7H). Before the age of nine, the foot progression angle increased externally with baseline age, while above the age of nine this angle increased internally.

## Discussion

This mixed cross-sectional longitudinal study aimed at providing a full description of the progressive gait pathology in children with DMD. First, 31 predefined gait features were cross-sectionally compared between children with DMD at baseline and a TD database. This allowed for a comparison of our results with previous literature. We hypothesized that the predefined gait features of children with DMD would deviate from TD children already at baseline, since the children with DMD enrolled at different ages, ranging from 4.6 to 15 years old, and therefore, presented different stages of the disease progression. Second, the longitudinal changes in the 31 predefined gait features in the same cohort of boys with DMD were explored in a follow-up study over a period of 5 years. We hypothesized that the predefined gait features would evolve significantly over time, as children with DMD grow older.

### Aim 1: Gait Features of Children With Duchenne Muscular Dystrophy at Baseline Compared to Typically Developing Children

Our hypothesis concerning the first aim was only partly confirmed, since 17 out of the 31 gait features differed in the boys with DMD at baseline compared to the TD children and not all differences were in agreement with previous findings. In comparison to the most commonly reported gait features summarized in the systematic review of Goudriaan et al. (2018a), we only agreed on a reduced walking velocity, step length, maximal dorsiflexion moment, and maximal ankle power generation in children with DMD compared to TD children. In addition, we found that boys with DMD walked with an increased anterior pelvic tilt in combination with a decreased hip extension at the end of stance, which is in agreement with previous findings (Sutherland et al., 1981; Boccardi et al., 1997; Gaudreault et al., 2010; Goudriaan et al., 2018b). At the beginning of the stance phase, the maximal hip extension moment was reduced in the boys with DMD compared to the TD children. This is in line with previous literature (Boccardi et al., 1997; D’Angelo et al., 2009; Gaudreault et al., 2010) and is explained by a smaller ground reaction force and reduced lever arm due to the more posteriorly aligned ground reaction force (Gaudreault et al., 2010). This is potentially achieved by backward trunk leaning commonly observed in children with DMD and suggested to be a compensation strategy for hip extensor weakness (Sutherland et al., 1981). In contrast to Boccardi et al. (1997), we found that the maximal hip flexion moment, later in stance, was also decreased in children with DMD, probably due to the increased maximal anterior pelvic tilt and decreased maximal hip extension at terminal stance. Furthermore, the boys with DMD walked with a broader base of support, which is consistent with previous literature and is considered a compensation mechanism for the weak hip abductors (Sutherland et al., 1981; Boccardi et al., 1997; Romano et al., 2019). The increased maximal external foot progression angle found in the children with DMD also aligned the ground reaction force more laterally. However, the maximal hip abduction moment was not reduced in DMD, which is in contrast with the findings reported by Gaudreault et al. (2010). The minimal knee flexion angle at the end of the stance phase was increased, while previous research more frequently reported knee hyperextension, as a compensation mechanism for weak knee extensors (D’Angelo et al., 2009; Doglio et al., 2011). In relation to the more flexed knee position at the end of stance, the range of knee flexion/extension motion during stance was decreased in DMD compared to TD. The maximal knee flexion moment was decreased in the boys with DMD. The compensation mechanism around the hip may also indirectly affect the kinetics around the knee, as the more posteriorly directed ground reaction force is probably located more closely to the knee joint. In contrast to the equinus gait commonly seen in clinical practice, ankle kinematics did not significantly differ between DMD at baseline and TD. However, the ankle kinetics showed some significant characteristics of an equinus walking pattern as the maximal dorsiflexion moment, maximal plantar flexion moment and maximal ankle power generation were reduced in DMD. In swing, boys with DMD presented an increased maximal hip flexion, maximal hip abduction and maximal knee flexion angle, which have been reported as compensation mechanisms for a drop foot, i.e., a lack of ankle dorsiflexion in swing (Sutherland et al., 1981; D’Angelo et al., 2009; Doglio et al., 2011).

In summary, our findings are not fully consistent with previous literature. Especially, knee hyperextension and equinus gait in the ankle kinematics were not detected. Since DMD is a progressive disorder, wide age ranges result in heterogeneous groups with boys that are located in different stages of the disease progression. Consequently, it is likely that the full spectrum of gait pathology is leveled out when such a heterogeneous group is compared to a TD group. Therefore, longitudinal analyses studying how gait features evolve over time in the same children with DMD are more of interest than investigating an average/mean of gait features in a heterogeneous group. This longitudinal description is also clinically relevant for treatments aiming to prolong ambulation.

### Aim 2: Evolution of Gait Features in Growing Boys With Duchenne Muscular Dystrophy

Our hypothesis concerning the second aim was confirmed. Indeed, several gait features showed a significant altered evolution in the boys with DMD. To avoid confusion since the cross-sectional baseline age-effect could be misinterpreted as a longitudinal time effect, both effects were discussed separately.

#### Longitudinal Effect

Twenty-one gait features evolved longitudinally. Overall, our results are in agreement with the changes summed over the three stages as reported by Sutherland et al. (1981).

##### Global Gait Outcome

The increase in GPS confirmed the overall progressive gait pathology seen in children with DMD. To our knowledge, the current study is the first in finding a significant progression in a global gait outcome. Previously reported differences in GPS and gait deviation index between progressive groups, which were classified based on motor abilities or the gait deviation index, did not reach significance (Sienko Thomas et al., 2010; de Souza et al., 2019). Mainly at older ages, a steep increase in GPS could be detected, while in young boys with DMD the increase in GPS was small. Therefore, the GPS might not be sensitive enough to detect altered gait pathology and thus relevant improvements in clinical trials, especially in young boys with DMD.

##### Sagittal and Transversal Pelvic Kinematics—Sagittal Hip Kinematics—Step Length

In agreement with Sutherland et al. (1981), the anterior pelvic tilt increased over time in the children with DMD (Supplementary Figure 1), potentially due to increasing stiffness and shortening of the hip flexors and/or hip extensor weakness (Boccardi et al., 1997; D’Angelo et al., 2009; Gaudreault et al., 2010). Consequently, the hip extension deficit increased by almost two degrees per year. To compensate for this and to improve step length (Doglio et al., 2011), the boys with DMD increased the motion of the pelvic rotation in the transverse plane over time. However, this compensation mechanism was not sufficient enough to prevent the decrease in step length.

##### Sagittal Hip Kinetics

The maximal hip extension moment and power generation decreased longitudinally in the boys with DMD, suggesting a longitudinal progression of the compensation for the weak hip extensor muscles. Unexpectedly, the steep decline in these gait features was first preceded by a small increase or stabilization in approximately the first 6–12 months of the follow-up period. Taking into account the results of Heberer et al. (2016), the start of corticosteroids or clinical trial participation may contribute to the initial improvements in the maximal hip extension moment and power generation (Heberer et al., 2016). This suggests that these two gait features may potentially monitor the effect of clinical trials that aim to prolong walking ability (Heberer et al., 2016).

##### Step Width—Frontal Pelvic and Hip Kinematics

Our longitudinal analysis revealed an initial small gait maturation toward a smaller step width in the boys with DMD (Sutherland, 1997; Sala and Cohen, 2013), followed by a steep longitudinal increase in step width after the age of nine (Supplementary Figure 2). Unexpectedly, a longitudinal increase in the maximal hip abduction was already visible from the start of the follow-up. This could be related to the observed longitudinal increase in range of motion of the pelvic obliquity, as a result of the previously reported contralateral elevation of the pelvis at the end of the stance phase (Gaudreault et al., 2010; Romano et al., 2019). In short, children with DMD present a complex progressive interaction between mechanisms to compensate for hip abductor weakness, i.e., increase in step width and pelvic obliquity.

##### Frontal Hip Kinetics

The longitudinal compensation mechanisms for hip abductor weakness were not reflected in a reduction of the maximal hip abduction moment. In fact, initially, the maximal hip abduction moment increased. Hip abductors have been reported as muscles that are likely to stiffen and contract in DMD (McDonald et al., 1995; Skalsky and McDonald, 2012). Therefore, it is expected that the passive forces, caused by the increased resistance to elongation of the stiff hip abductors, contribute to the net moments during gait (Gaudreault et al., 2009). Since several hip abductors also act as internal hip rotators (Neumann, 2010), stiff and contracted hip abductors may have resulted in the increasing internal foot progression. This may explain the increase in the maximal hip abduction moment, since the ground reaction force is more medially located to the hip joint with internally rotated feet. Only after the age of nine, the maximal hip abduction moment started to decrease longitudinally, suggesting that the beneficial effect of the stiffness and contracture may no longer sufficiently compensate the weak hip abductors. Therefore, boys with DMD older than nine years would need to use other compensation mechanisms, such as increasing the step width, to align the ground reaction force more laterally, which was indeed observed in the current study.

##### Knee Kinematics

The maximal knee flexion angle during loading response decreased longitudinally. Avoiding knee flexion to compensate for knee extensor muscle weakness has been reported in previous cross-sectional research comparing the gait features between DMD and TD (D’Angelo et al., 2009; Romano et al., 2019). The range of knee flexion/extension motion during stance first increased and then decreased longitudinally. Interestingly, Doglio et al. (2011) reported an increased knee range of motion due to knee hyperextension at the end of stance in young boys with DMD compared to TD children (Doglio et al., 2011). Furthermore, a decrease in the maximal knee extension angle at the end of stance, possibly due to the formation of knee flexion contractures in a later stage of the disease (Sussman, 2002; Choi et al., 2018), may contribute to a reduction in knee flexion/extension range of motion. In the current study, however, no significant average evolution toward knee hyperextension could be detected. Yet, looking at the individual predicted profiles, these gait features seemed to be related. In boys with DMD progressing toward an increased maximal knee extension at the end of stance, the knee range of motion increased and vice versa.

##### Knee Kinetics

Although the maximal knee flexion angle during loading response decreased longitudinally, there was no change in the maximal knee extension moment. Therefore, our findings could not confirm the longitudinal progression of the compensation for weakness in the knee extensors. On a patient-specific level, both increasing as well as decreasing evolutions in the maximal knee extension moment were observed. Hence, it might be that some of the boys with DMD appeared to have sufficient knee extensor strength to control the increasing maximal knee extension moment. This might have been a requirement for ambulation, since a larger knee extension moment is difficult to avoid with the previously described compensation around the hip (i.e., posterior alignment of the ground reaction force due to backward trunk leaning). Moreover, this clinical presentation of these boys with DMD matched with limited gait pathology in the early stage as described by Sutherland et al. (1981). Others, who corresponded more to the transitional stage of Sutherland et al. (1981), needed to compensate for increasing knee extensor weakness by decreasing the knee extension moment. Due to the progressive muscle weakness, a switch from an increasing toward a decreasing knee extension moment was expected. However, such a transition, from the early to the transitional stage, was not captured in the current longitudinal analyses of the same children with DMD. The maximal knee flexion moment presented first a small increase or stabilization, which was followed by a steep decline longitudinally. Therefore, the decreasing knee flexion moment may be an indirect result of the increasing compensation strategy for weak hip extensors (i.e., posterior alignment of the ground reaction force to the hip joint results in a closer alignment of the ground reaction force to the knee joint). In summary, the longitudinal evolution in the maximal knee extension and flexion moments were thus less straightforward, most likely due to the influence of the proximal compensations and the presence of different gait stages across patients.

##### Ankle Kinematics

The current study partly confirmed the progressive equinus gait reported by Sutherland et al. (1981). An evolution from a normal heel strike to flatfeet followed by forefoot contact was captured, which is probably caused by muscle weakness of the tibialis anterior and/or muscle contracture of the gastrocnemius (Sussman, 2002; Gaudreault et al., 2009; Yiu and Kornberg, 2015). Additionally, the observed increased plantar flexion angle resulted in a ground reaction force that was more anteriorly to the knee joint, and may serve as well as a compensation mechanism for knee extensor weakness (Sutherland et al., 1981; D’Angelo et al., 2009; Doglio et al., 2011). But, in the current study, there was no average longitudinal reduction in the maximal dorsiflexion angle during stance. The onset and worsening of a midfoot break may have contributed to a dorsiflexion overestimation in some of the boys with DMD. Yet, based on the plantar flexion angle at initial contact and the video recordings detecting a midfoot break in combination with a premature heel rise, the longitudinal increase in equinus gait in the children with DMD was evident.

##### Ankle Kinetics

The progressive equinus gait could only be partly confirmed. Due to the progression toward flat- or forefeet strike, the maximal dorsiflexion moment reduced longitudinally. No significant longitudinal evolution was detected for the maximal plantar flexion moment and ankle power generation. This suggests that the progressive weakness of the plantar flexors may have limited impact on the ankle kinetics during push-off. However, similar as suggested for the maximal dorsiflexion angle during stance, the presence of a midfoot break might have influenced the lever arm and thus the ankle kinetics during push-off. Therefore, characteristics of a progressive equinus gait in the ankle kinetics were mainly visible during loading response, when no midfoot break was present.

##### Swing Phase

During swing, the ankle progressed toward a drop foot over time in the boys with DMD (Supplementary Figure 3), which is in agreement with the changes over the three stages reported by Sutherland et al. (1981). Increasing the maximal hip abduction angle, the maximal knee flexion angle, and the maximal hip flexion angle have been reported as compensation mechanisms to aid foot clearance and avoid tripping (Sutherland et al., 1981; D’Angelo et al., 2009; Doglio et al., 2011). Similar longitudinal evolutions in the maximal dorsiflexion angle and the maximal hip flexion angle in swing were found, i.e., the longitudinal decrease in the maximal dorsiflexion angle seemed to be related with the longitudinal increase in the maximal hip flexion angle, both progressing more rapidly with increasing baseline age. Therefore, the current study suggests that the evolution in the maximal hip flexion angle in swing could be the main compensation for the progressive drop foot.

#### Cross-Sectional Baseline Age-Effect

Since DMD is a progressive disorder, an increase or decrease in the gait features with age was expected. This was confirmed by the current study, as 25 gait features presented a significant cross-sectional baseline age-effect. Regarding the age-effect, the evolution in several gait features (e.g., GPS, maximal anterior pelvic tilt, range of pelvic motion, maximal hip extension moment, maximal hip power, maximal plantar flexion angle in stance, and swing) was often preceded by an opposite trend. Since boys with DMD achieve independent walking generally at a later age compared to TD (Sussman, 2002), the younger boys with DMD may still present an immature gait pattern. Therefore, this opposite trend in the gait features may represent a maturation process. Similar to Heberer et al. (2016), it is also possible that starting the use of corticosteroids or a novel therapeutic strategy in a clinical trial may result in an improvement in the gait features. However, the effect of baseline age on the gait features is a cross-sectional analysis and therefore, the variability among patients could be misinterpreted as a maturation or treatment effect. Additionally, some of the gait features presented an opposite cross-sectional baseline age-effect to the longitudinal effect. For example, the maximal knee flexion angle decreased longitudinally, but, increased with the cross-sectional baseline age. This baseline age-effect could be explained by the absence and presence of a knee flexion contracture, which was measured during a standard clinical exam (goniometry), in the younger and older boys with DMD, respectively. However, the progression from decreasing the maximal knee flexion angle to compensate for knee extensor weakness toward increasing the maximal knee flexion angle when a knee flexion contracture starts to develop was not detected within the same patients. Therefore, the cross-sectional age-effect may reflect the heterogeneity among children with DMD instead of a longitudinal evolution.

### Limitations

Although the current study investigated a unique mixed longitudinal database, the amount of 3DGA-sessions collected over the entire ambulation period in the same boys with DMD was still limited. To take into account that the children with DMD who enrolled at older ages were in more advanced stages of the disease, the effect of increasing baseline age was studied. Due to the small follow-up period in some of the children (i.e., minimal follow-up time of 6 months), the longitudinal effect was not always in agreement with the cross-sectional effect of increasing baseline age. Therefore, caution is needed with the interpretation of the baseline age-effect, since it could be partly influenced by the existing variation between patients. Additionally, the differences in clinical background such as the underlying gene mutation, clinical trial participation, corticosteroid doses, periods of serial casting, and the functional level, etc.^1^ among the boys with DMD, may have contributed to the heterogeneity of our study sample. Furthermore, several gait features were solely defined based on the maximal or minimal value. Therefore, the timing of this value in the gait cycle could vary between as well as within the boys with DMD depending on how pathological the gait pattern was at a certain stage of the disease progression. Statistical analysis, such as statistical (non-)parametric mapping, could be used to take into account the time dependency. However, LMM are not yet implemented in statistical (non-)parametric mapping. Lastly, our simplistic foot model (Plug-In Gait Full-Body model) could have resulted in a dorsiflexion overestimation, if the boys with DMD presented a midfoot break.

## Conclusion

In conclusion, we provided a quantitative description of the evolution in gait features in growing boys with DMD based on a unique mixed longitudinal dataset and therefore, our findings improved the understanding of the natural history of the progressive DMD gait pathology. Despite the limited follow-up period and the large variability between the children with DMD, we found that 21 gait features progressed longitudinally in the patients with DMD. This suggests that these gait features are promising outcome measures for future clinical trials to quantify the efficacy of novel therapeutic strategies aiming to prolong ambulation. Next steps are (1) the assessment of the sensitivity of these gait features to change (e.g., in clinical trials) and comparing them to the outcomes from standard clinical measurements (e.g., 6MWT and NSAA), (2) the confirmation of our findings and the investigation of the effect of treatments (i.e., corticosteroid use, clinical trial participation and serial casting) as well as the effect of individual characteristics (e.g., underlying gene mutation and functional level) on the gait evolution in a larger sample size with a longer follow-up per participant and a larger presentation of boys in the late stage of the disease and, (3) the exploration of how the underlying impairments, e.g., progressive muscle weakness and stiffness, contribute to the progressive gait pathology.

## Data Availability Statement

The datasets presented in this study can be found in online repositories. The names of the repository/repositories and accession number(s) can be found in the article/Supplementary Material.

## Ethics Statement

The studies involving human participants were reviewed and approved by the local ethics committee (Ethical Committee UZ Leuven/KU Leuven; S55867, S56041, and S61324). Written informed consent to participate in this study was provided by the participants’ legal guardian/next of kin.

## Author Contributions

IV and KD: conceptualization. IV, MVdH, NDB, MG, and ES: data curation. IV, MD, and GM: formal analysis. IV, NG, LDW, and KD: funding acquisition. IV, MD, GM, and KD: methodology. KD: resources and supervision. IV: visualization and writing–original draft. MVdH, NDB, ES, MG, MD, GM, NG, LDW, AVC, FDG, and KD: writing–review and editing. All authors have read and agreed to the published version of the manuscript.

## Conflict of Interest

The authors declare that the research was conducted in the absence of any commercial or financial relationships that could be construed as a potential conflict of interest.

## Publisher’s Note

All claims expressed in this article are solely those of the authors and do not necessarily represent those of their affiliated organizations, or those of the publisher, the editors and the reviewers. Any product that may be evaluated in this article, or claim that may be made by its manufacturer, is not guaranteed or endorsed by the publisher.

## Footnotes

1
https://dx.doi.org/10.6084/m9.figshare.17313188
