# Supplementary material for: Longitudinal Alterations in Gait Features in Growing Children With Duchenne Muscular Dystrophy
Source: Front Hum Neurosci. 2022 Jun 2;16:861136. doi: 10.3389/fnhum.2022.861136 (PMC9201072; doi:10.3389/fnhum.2022.861136)
Supplement: Supplementary file 1 [file Data_Sheet_1.docx]

**Supplementary table 1**: 95% confidence intervals of fixed effects’ estimates of the linear mixed effect models with spatiotemporal parameters, kinematics and kinetics as responses in children with DMD.

|  |  | Fixed effects | | | | | | | | | | | | |  |
| --- | --- | --- | --- | --- | --- | --- | --- | --- | --- | --- | --- | --- | --- | --- | --- |
|  |  | Intercept |  | Regression coefficients | | | | | | | | | | |  |
|  |  |  |  | Longitudinal effects | | |  | Cross-sectional effects | | | | |  | Interaction effects | |
|  |  |  |  | Time |  | Time^2^ |  | Baseline age |  | Baseline age^2^ |  | Baseline age^3^ |  | Time* Baseline age | |
| Variables |  | β_0_ |  | β_1_ |  | β_2_ |  | β_3_ |  | β_4_ |  | β_5_ |  | β_6_ | |
| Cadence(steps/s) |  | 2.464 - 2.770 |  | -0.092 - -0.023 |  |  |  | -0.185 - -0.063 |  | 0.001 - 0.011 |  |  |  |  | |
| WVnorm(/) |  | 0.423 - 0.492 |  | -0.024 - 0.001 |  |  |  | -0.017 - -0.006 |  |  |  |  |  |  | |
| SLnorm(/) |  | 0.745 - 0.826 |  | -0.030 - -0.003 |  |  |  | -0.015 - -0.003 |  |  |  |  |  |  | |
| SWnorm(/) |  | 0.234 - 0.290 |  | -0.042 - -0.012 |  | 0.002 - 0.008 |  | -0.003 - 0.009 |  |  |  |  |  | 0.003 - 0.007 | |
| Gait profile score(°) |  | 5.28 - 8.10 |  | -0.32 - 0.70 |  |  |  | -1.21 - 0.07 |  | 0.02 - 0.16 |  |  |  | 0.01 - 0.20 | |
| Max anterior pelvic tilt(°) |  | 11.63 - 19.78 |  | 0.66 - 2.00 |  |  |  | -3.03 - 0.20 |  | 0.12 - 0.40 |  |  |  |  | |
| ROM pelvic obliquity(°) |  | 5.91 - 9.28 |  | 0.24 - 0.92 |  |  |  | 0.34 - 1.14 |  |  |  |  |  |  | |
| ROM pelvic rotation(°) |  | 10.10 - 16.54 |  | 0.14 - 2.56 |  |  |  | -3.04 - 0.51 |  | 0.07 - 0.44 |  |  |  |  | |
| Min hip flex angle stance(°) |  | -12.13 - -3.13 |  | 0.74 - 3.02 |  |  |  | -2.08 - 1.64 |  | 0.05 - 0.41 |  |  |  |  | |
| Max hip flex angle swing(°) |  | 32.36 - 41.85 |  | -1.44 - 0.99 |  |  |  | 0.30 - 1.86 |  |  |  |  |  | 0.09 - 0.50 | |
| ROM hip sagittal plane(°) |  | 42.93 - 51.27 |  | -1.57 - 0.36 |  |  |  | -1.44 - -0.09 |  |  |  |  |  |  | |
| Max hip ext mom stance(Nm/kg) |  | 0.384 - 0.621 |  | -0.037 - 0.106 |  | -0.035 - -0.005 |  | 0.013 - 0.116 |  | -0.015- -0.005 |  |  |  |  | |
| Max hip power stance(W/kg) |  | 0.270 - 0.680 |  | -0.060 - 0.193 |  | -0.061 - -0.0002 |  | -0.010 - 0.133 |  | -0.015 - -0.002 |  |  |  |  | |
| Min hip add angle stance(°) |  | -5.34 - -1.29 |  | -0.79 - -0.05 |  |  |  | -2.03 - -0.44 |  | 0.02 - 0.17 |  |  |  |  | |
| Min hip add angle swing(°) |  | -8.99 - -4.85 |  | -1.04 - 0.09 |  |  |  | -2.25 - -0.58 |  | 0.05 - 0.19 |  |  |  |  | |
| Max hip abd mom stance(Nm/kg) |  | 0.445 - 0.628 |  | 0.005 - 0.052 |  |  |  | 0.002 - 0.031 |  |  |  |  |  | -0.016 - -0.004 | |
| Max knee flex angle stance(°) |  | 29.75 - 34.53 |  | -1.35 - -0.36 |  |  |  | 0.18 - 1.28 |  |  |  |  |  |  | |
| Min knee flex angle stance(°) |  | 1.43 - 6.04 |  | -1.49 - 0.51 |  |  |  | 0.45 - 1.54 |  |  |  |  |  |  | |
| ROM knee sagittal plane stance(°) |  | 23.02 - 29.09 |  | -0.67 - 1.89 |  | -0.59 - -0.12 |  | -0.29 - 2.34 |  | -0.24 - -0.01 |  |  |  |  | |
| Max knee flex angle swing(°) |  | 66.60 - 71.05 |  | -2.06 - -0.44 |  |  |  |  |  |  |  |  |  |  | |
| Max knee ext mom stance(Nm/kg) |  | 0.363 - 0.473 |  | -0.052 - 0.017 |  |  |  |  |  |  |  |  |  |  | |
| Min knee ext mom stance(Nm/kg) |  | -0.114 - 0.028 |  | -0.045 - 0.011 |  | 0.002 - 0.014 |  | -0.129 - -0.007 |  | 0.004 - 0.036 |  | -0.002 - -0.0002 |  |  | |
| Dorsiflex angle IC(°) |  | -2.31 - 5.70 |  | -1.72 - -0.67 |  |  |  | -1.06 - 2.10 |  | -0.29 - -0.01 |  |  |  |  | |
| Max dorsiflex angle stance(°) |  | 7.96 - 14.72 |  | -1.38 - 0.20 |  |  |  | 1.12 - 4.17 |  | -0.46 - -0.19 |  |  |  |  | |
| Max dorsiflex angle swing(°) |  | -2.77 - 7.05 |  | -1.01 - 1.13 |  |  |  | 0.33 - 3.84 |  | -0.42 - -0.15 |  |  |  | -0.54 - -0.17 | |
| Min plantar flex mom LR(Nm/kg) |  | -0.076 - -0.043 |  | 0.005 - 0.015 |  |  |  |  |  |  |  |  |  |  | |
| Max plantar flex mom PO(Nm/kg) |  | 0.792 - 0.943 |  | -0.036 - 0.017 |  |  |  | 0.023 - 0.051 |  |  |  |  |  |  | |
| Min ankle power LR(W/kg) |  | -0.760 - -0.307 |  | -0.112 - 0.061 |  |  |  | -0.041 - 0.159 |  | -0.020 - -0.002 |  |  |  |  | |
| Max ankle power PO(W/kg) |  | 2.587 - 3.164 |  | -0.214 - 0.163 |  |  |  |  |  |  |  |  |  |  | |
| Max int foot prog angle stance(°) |  | -10.13 - -2.46 |  | 0.85 - 3.36 |  |  |  | -4.96 - -1.15 |  | 0.18 - 0.62 |  |  |  |  | |

Abbreviations in alphabetic order: abd=abduction; add=adduction; DMD=Duchenne muscular dystrophy; dorsiflex=dorsiflexion; ext=extension; flex=flexion; IC=initial contact; int=internal; LR=loading response; Max=maximal; Min=minimal; mom=moment; PO=push-off; prog=progression; ROM=range of motion; SLnorm=normalized step length; SWnorm=normalized step width; WVnorm=normalized walking velocity

**Supplementary table 2**: Random-effect and residual covariance structure of the linear mixed effect models with spatiotemporal parameters, kinematics and kinetics as responses in children with DMD.

|  |  | Random effects | |  | Residual |  | Serial correlation sp(gau) | |
| --- | --- | --- | --- | --- | --- | --- | --- | --- |
|  |  | Variance random intercept | Variance random slope |  | Variance residual |  | Correlation | Variance |
| Variables |  | σ^2^( b_1i_) | σ^2^( b_2i_) |  | σ^2^( ε_(1)ij_) |  |  | σ^2^( ε_(2)ij_) |
| Cadence(steps/s) |  | 0.025 | 0.004 |  | 0.012 |  |  |  |
| WVnorm(/) |  | 0.002 | 0.001 |  | 0.001 |  |  |  |
| SLnorm(/) |  | 0.003 | 0.001 |  | 0.002 |  |  |  |
| SWnorm(/) |  | 0.001 |  |  | 0.001 |  | 1.39 | 0.001 |
| Gait profile score(°) |  | 1.39 |  |  | 1.91 |  |  |  |
| Max anterior pelvic tilt(°) |  | 10.38 | 1.63 |  | 4.45 |  |  |  |
| ROM pelvic obliquity(°) |  | 8.71 |  |  | 4.81 |  |  |  |
| ROM pelvic rotation(°) |  | 24.12 | 6.11 |  | 8.03 |  |  |  |
| Min hip flex angle stance(°) |  | 11.06 | 4.14 |  | 15.44 |  |  |  |
| Max hip flex angle swing(°) |  | 36.27 |  |  | 18.92 |  |  |  |
| ROM hip sagittal plane(°) |  | 30.59 | 3.55 |  | 6.26 |  |  |  |
| Max hip ext mom stance(Nm/kg) |  | 0.020 |  |  | 0.031 |  |  |  |
| Max hip power stance(W/kg) |  | 0.045 |  |  | 0.072 |  |  |  |
| Min hip add angle stance(°) |  | 4.66 |  |  | 4.70 |  |  |  |
| Min hip add angle swing(°) |  | 4.69 | 0.68 |  | 5.81 |  |  |  |
| Max hip abd mom stance(Nm/kg) |  | 0.016 |  |  | 0.012 |  |  |  |
| Max knee flex angle stance(°) |  | 8.66 |  |  | 14.71 |  |  |  |
| Min knee flex angle stance(°) |  | 10.38 | 2.77 |  | 12.84 |  |  |  |
| ROM knee sagittal plane stance(°) |  | 10.13 | 4.91 |  | 5.20 |  |  |  |
| Max knee flex angle swing(°) |  | 15.23 |  |  | 16.81 |  |  |  |
| Max knee ext mom stance(Nm/kg) |  | 0.013 | 0.004 |  | 0.011 |  |  |  |
| Min knee ext mom stance(Nm/kg) |  | 0.005 |  |  | 0.006 |  |  |  |
| Dorsiflex angle IC(°) |  | 18.47 |  |  | 6.52 |  | 0.94 | 6.77 |
| Max dorsiflex angle stance(°) |  | 11.70 | 1.86 |  | 7.17 |  |  |  |
| Max dorsiflex angle swing(°) |  | 27.36 |  |  | 10.11 |  |  |  |
| Min plantar flex mom LR(Nm/kg) |  | 0.001 |  |  | <0.001 |  | 0.81 | 0.001 |
| Max plantar flex mom PO(Nm/kg) |  | 0.015 | 0.002 |  | 0.007 |  |  |  |
| Min ankle power LR(W/kg) |  | 0.060 | 0.031 |  | 0.042 |  |  |  |
| Max ankle power PO(W/kg) |  | 0.404 | 0.145 |  | 0.224 |  |  |  |
| Max int foot prog angle stance(°) |  | 20.77 | 5.47 |  | 13.30 |  |  |  |

Abbreviations in alphabetic order: abd=abduction; add=adduction; DMD=Duchenne muscular dystrophy; dorsiflex=dorsiflexion; ext=extension; flex=flexion; IC=initial contact; int=internal; LR=loading response; Max=maximal; Min=minimal; mom=moment; PO=push-off; prog=progression; ROM=range of motion; SLnorm=normalized step length; SWnorm=normalized step width; WVnorm=normalized walking velocity

.
