# Supplementary material for: Longitudinal Alterations in Gait Features in Growing Children With Duchenne Muscular Dystrophy
Source: Front Hum Neurosci. 2022 Jun 2;16:861136. doi: 10.3389/fnhum.2022.861136 (PMC9201072; doi:10.3389/fnhum.2022.861136)
Supplement: Supplementary file 2 [file Data_Sheet_2.docx]

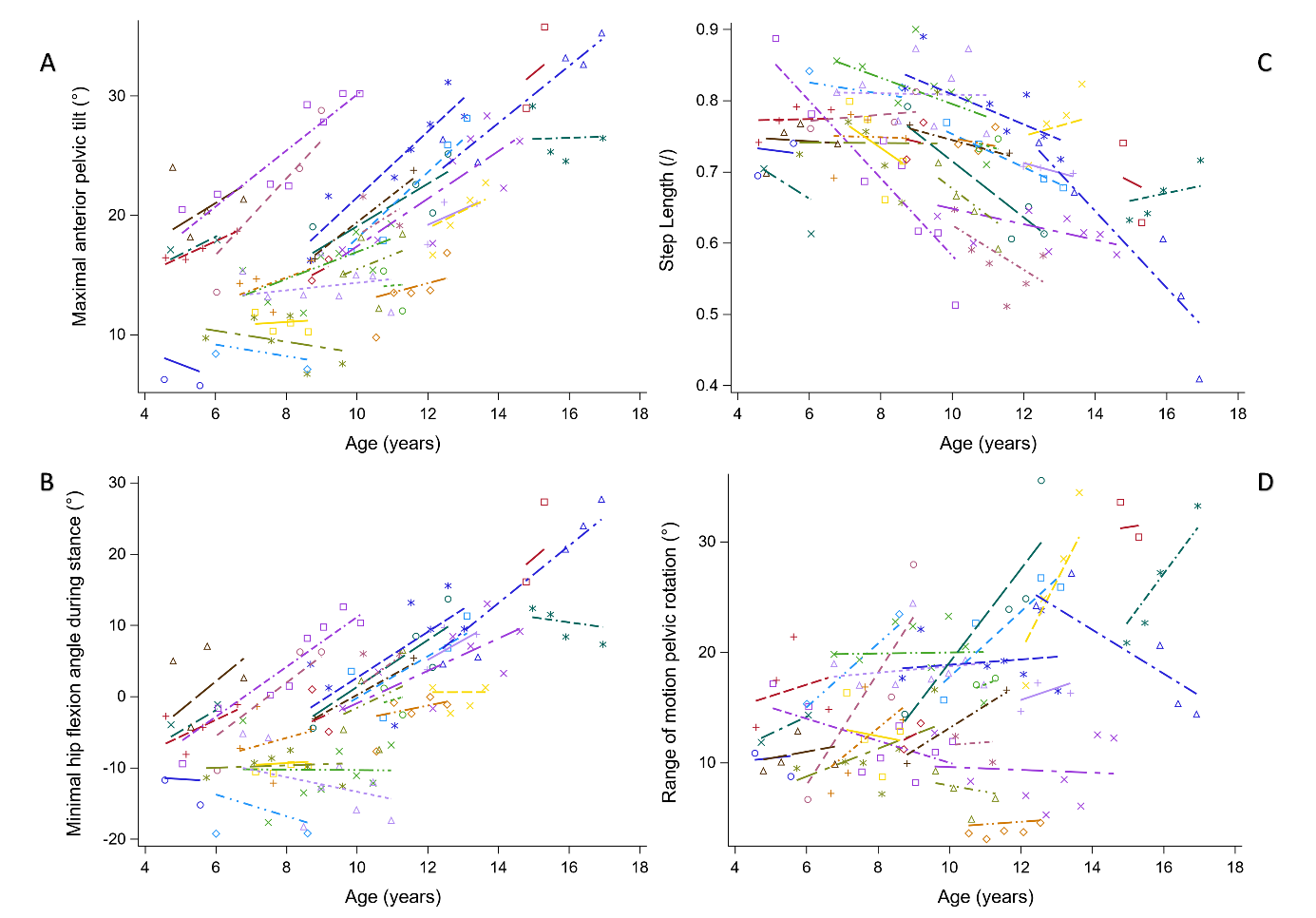


**Supplementary figure 1** The individual predicted profiles (dashed lines) for the maximal anterior pelvic tilt **(A)**, the minimal hip flexion angle during stance **(B)**, normalized step length **(C)**, range of motion pelvic rotation **(D).** The actual observed values are visualized by the symbols. Each color represents one patient with DMD. The regression coefficients of the fixed effects are given in Tables 3-5.

Abbreviations in alphabetic order: DMD=Duchenne muscular dystrophy;


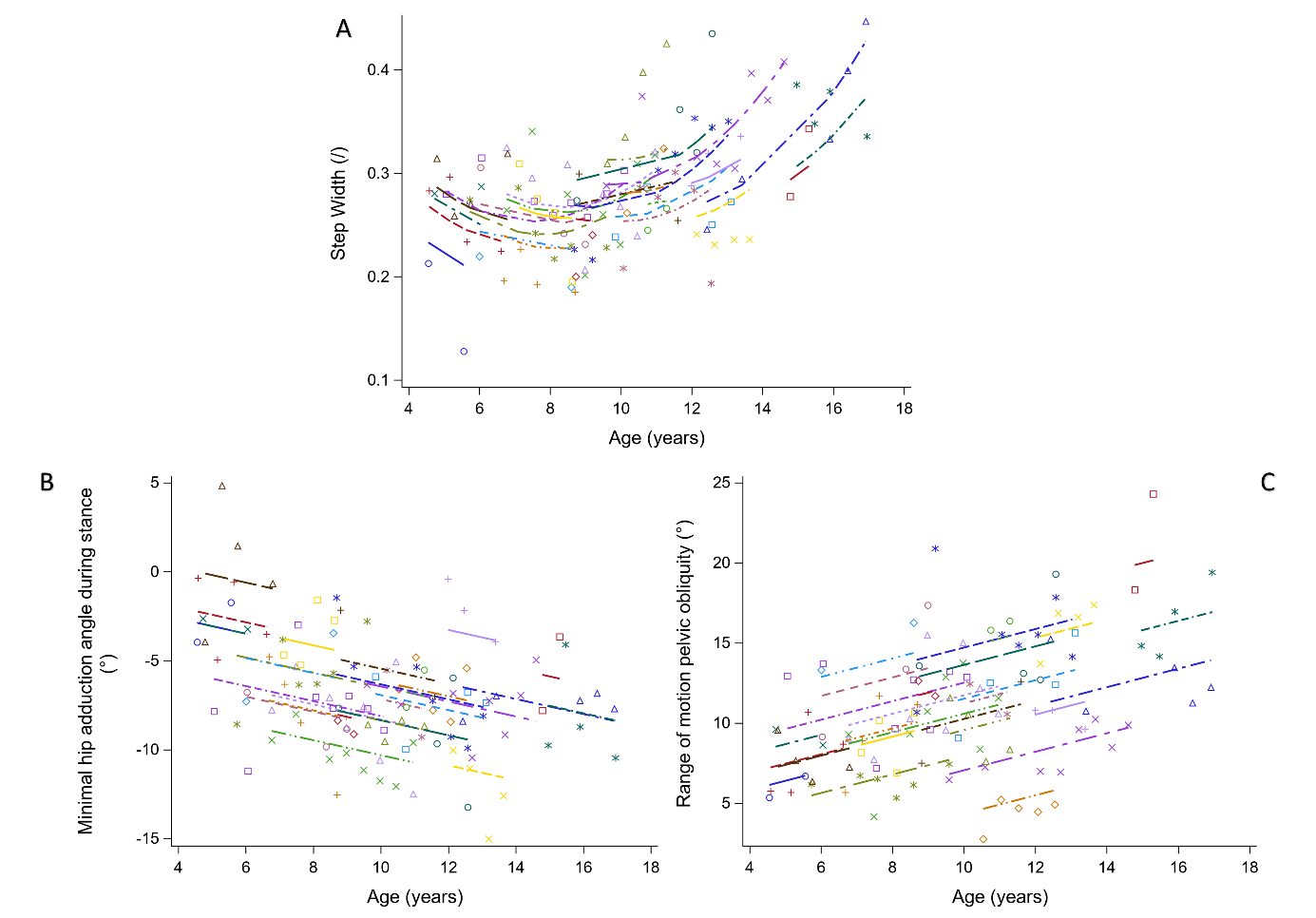


**Supplementary figure 2** The individual predicted profiles (dashed lines) for the normalized step width **(A)**, the minimal hip adduction angle during stance **(B)**, range of motion pelvic obliquity **(C).** The actual observed values are visualized by the symbols. Each color represents one patient with DMD. The regression coefficients of the fixed effects are given in Tables 3-5.

Abbreviations in alphabetic order: DMD=Duchenne muscular dystrophy;


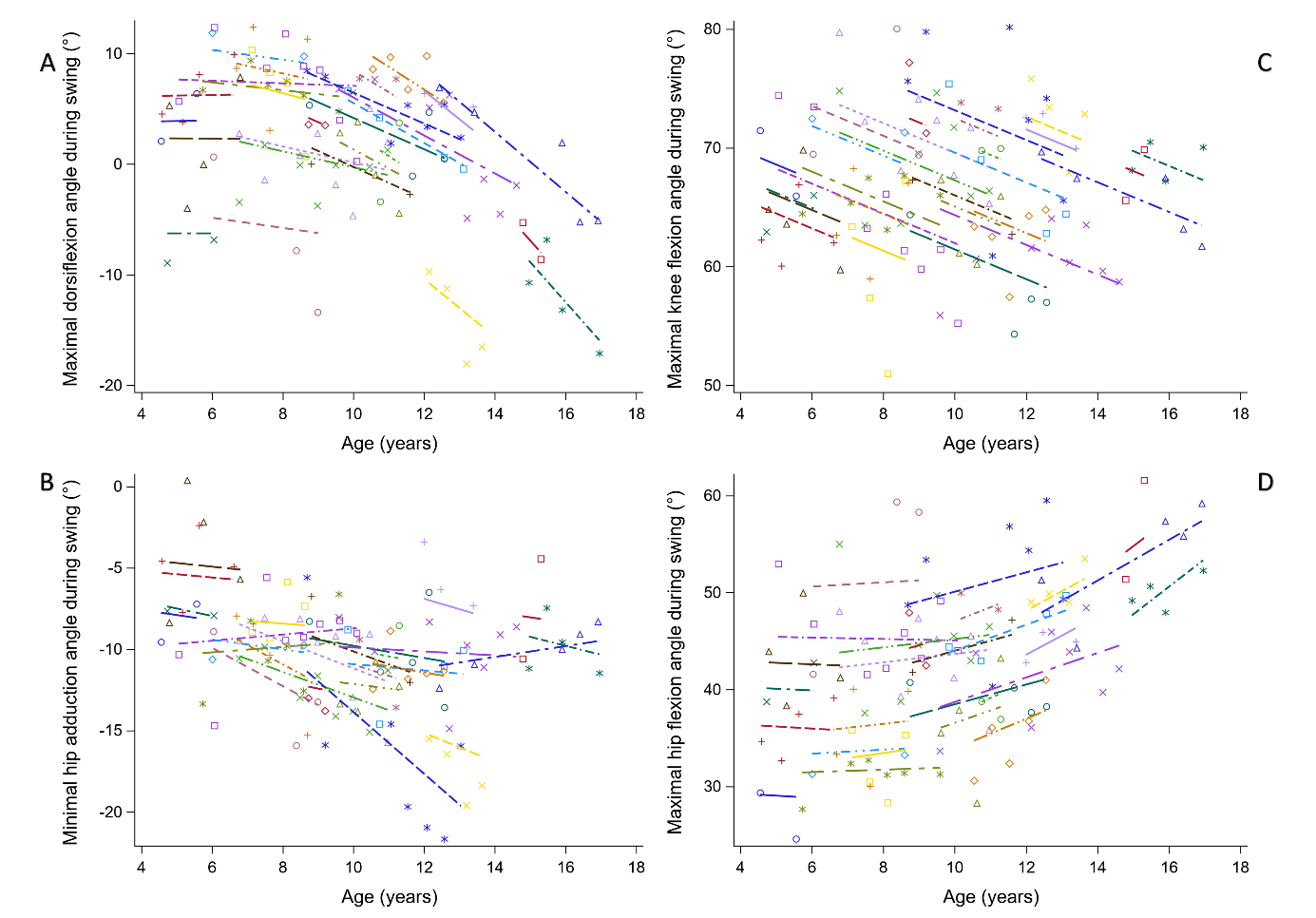


**Supplementary figure 3** The individual predicted profiles (dashed lines) for the maximal dorsiflexion angle during swing **(A)**, the minimal hip adduction angle during swing **(B)**, the maximal knee flexion angle during swing **(C)**, maximal hip flexion angle during swing **(D).** The actual observed values are visualized by the symbols. Each color represents one patient with DMD. The regression coefficients of the fixed effects are given in Tables 5-7.

Abbreviations in alphabetic order: DMD=Duchenne muscular dystrophy;
